# Community-level social capital and participation in health checkups among older adults in Japan: a cross-sectional study

**DOI:** 10.3389/fpubh.2025.1697600

**Published:** 2026-01-14

**Authors:** Hitomi Matsuura, Yoko Hatono

**Affiliations:** 1 Department of Health Sciences, Faculty of Medical Sciences, Kyushu University, Fukuoka, Japan; 2 Department of Nursing Faculty of Nursing and Human Nutrition, Yamaguchi Prefectural University, Yamaguchi, Japan; 3 Department of Public Health Nursing, Graduate Program, Kumamoto Health Sciences University, Kumamoto, Japan

**Keywords:** cross-sectional study, logistic regression analysis, medical checkup, older adult, social capital, social cohesion

## Abstract

**Background:**

Community-level social capital has been suggested as a contextual factor influencing preventive health behaviors among older adults, yet empirical evidence from micro-scale subcommunity settings remains limited. This study examined whether community-level social capital is associated with participation in specific medical checkups among older adults living in a typical regional city in Japan.

**Methods:**

A cross-sectional survey was conducted among 13,558 adults aged 65–74 years in Uwajima City. Community-level cognitive social capital was assessed using three subscales: civic participation, social cohesion, and reciprocity. Multilevel logistic regression analysis was performed across 65 voting districts, adjusting for individual lifestyle factors, socioeconomic status, and individual-level social capital.

**Results:**

After adjusting for covariates, including individual-level social capital, a one–standard deviation increase in community-level social cohesion was significantly associated with higher odds of participation in specific medical checkups (odds ratio per standard deviation increase: 1.11; 95% confidence interval: 1.03–1.19).

**Conclusion:**

Community-level social cohesion independently contributes to increased participation in preventive health checkups among older adults. These findings highlight the potential value of subcommunity-level strategies to promote preventive health behaviors in aging populations.

## 1 Introduction

Rapid population aging is a global phenomenon. According to the World Health Organization, the number of people aged 60 years and older worldwide is projected to grow from about 1 billion in 2020 to 1.4 billion by 2030, and the global population of people aged 60 years and above is expected to double to approximately 2.1 billion by 2050 (1). Such a rapid demographic shift has far-reaching implications, including an increase in lifestyle-related diseases and a substantial rise in healthcare expenditures. In response, many countries, including Japan, have emphasized preventive care and early detection through regular health checkups (2). In Japan, specific medical checkups (3) are conducted for all individuals aged 40–74 years to prevent and detect lifestyle-related diseases, such as hypertension, diabetes, and dyslipidemia. These checkups are designed to prevent lifestyle-related diseases and frailty and are generally free or heavily subsidized. However, despite their importance, participation remains suboptimal, with a rate of only 37.5% among NHI enrollees as of 2022 (4). Improving participation in these checkups has thus become a pressing public health priority both in Japan and globally.

Previous studies have shown that participation in regular health checkups is associated with lower mortality (5, 6) and reduced medical costs (7). At the individual level, factors such as educational attainment, economic status, and health perception influence participation (8–10). Additionally, studies have shown that individuals who undergo medical checkups tend to receive more social support (11) and capital (12, 13) than participants who do not. Social capital—defined as “features of social organization such as trust, norms, and networks that facilitate coordinated action” (14)—has been linked to behaviors including increased physical activity (15, 16), reduced smoking (17, 18), and moderation in alcohol consumption (19, 20). However, most of these studies have focused on individual-level social capital, while the influence of community-level social capital on participation in health checkups remains underexplored. Only a few studies, such as one conducted in Denmark (21), have examined the association between community social capital and voluntary medical checkups, and these studies primarily targeted adults aged 25–65 years and employed limited, one-dimensional measures of social capital.

Recent international studies have emphasized the importance of not only individual-level but also community-level social capital in shaping preventive health behaviors. For example, a multilevel study conducted in South Korea (22) reported that higher levels of regional trust, a cognitive form of social capital, were significantly associated with greater participation in national health screenings, even after adjusting for individual socioeconomic factors. In the United States, a study (23) demonstrated that counties with stronger community cohesion and higher institutional trust exhibited notably higher mammography screening uptake among older women. Evidence from China further supports this pattern: another study (24) found that middle-aged and older adults residing in communities with more opportunities for social interaction were more likely to undergo routine health examinations.

However, such findings are not entirely consistent across all contexts. In Japan, Takahashi and Nakao (25) analyzed municipality-level data and reported that factors used as proxies for social capital did not show a clear independent association with colorectal cancer screening once demographic and household structure variables were controlled for. Similarly, Kobayashi et al. (26) found that while caregivers’ individual-level social capital was significantly associated with children’s participation in regular dental checkups, community-level social capital showed no significant effect. These mixed results suggest the need to reconsider the level of aggregation of the geographic units used to operationalize social capital. In particular, conventional units such as municipalities or school districts may be too broad or socially diffuse to adequately capture the neighborhood-level influences that are likely to be most salient for older adults.

Theoretically, social capital comprises both structural and cognitive dimensions (5), and these two components may influence participation in specific health checkups. Structural social capital, such as engagement in community organizations, may facilitate the dissemination of health-related information and the provision of mutual support for preventive behaviors. Cognitive social capital, including trust in others and a perceived sense of social cohesion, may enhance trust in public health activities and increase individuals’ willingness to undergo specific health checkups.

To address the existing gaps, this study examined the association between community-level social capital and participation in specific health checkups among older adults in Japan. Rather than using conventional school districts or municipalities, we adopted voting districts, smaller territorial units with a more tangible lived reality for older adults and within their typical walking range, as a proxy for community-level social capital, thereby capturing neighborhood contexts most relevant to older residents. This study makes three key contributions. First, it is the first to investigate this relationship among older Japanese adults using community units of a walkable scale. Second, it simultaneously evaluates both the structural and cognitive dimensions of social capital, addressing the limitations of previous one-dimensional measures. Third, it provides empirical evidence with potential policy implications for promoting participation in specific health checkups. Nonetheless, we acknowledge that the indicators of social capital are proxy measures and that causal interpretations are limited by the observational nature of the study design.

## 2 Materials and methods

### 2.1 Study sample

This was a cross-sectional, census-based survey conducted using a self-administered questionnaire targeting all residents aged 65–74 years living in Uwajima City, Ehime Prefecture (population as of April 2025: 66,234; 41.4% aged ≥65 years). Uwajima City was established in 2005 through the merger of one city and three towns and consists of 76 voting districts spanning mountainous, coastal, and urban areas. In Japan, based on installation criteria set by the Ministry of Internal Affairs and Communications, voting stations must be located within 3 km of residents’ homes and are therefore typically situated within walking distance. In Uwajima City, adults aged ≥65 years are eligible to receive specific health checkups free of charge.

**Figure 1:**
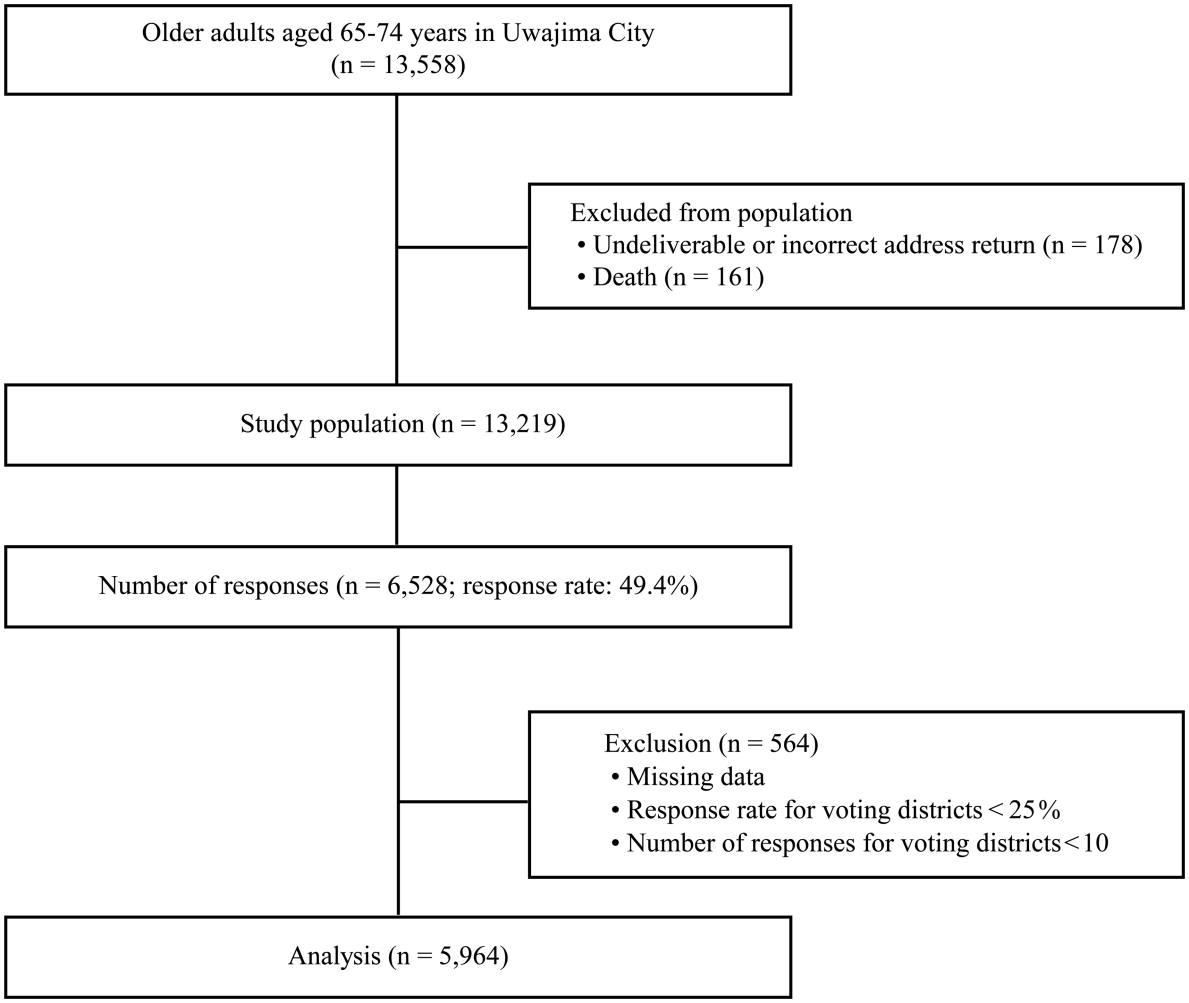
Flowchart of sample selection.

The study population comprised all 13,558 men and women aged 65–74 years residing in all 76 voting districts as of December 2017. The self-administered questionnaire was distributed by mail between 5 January and 9 February 2018. The participant selection process is illustrated in Figure 1.

Valid responses were those for which complete data on voting district, sex, and social capital indicators were available. For each voting district, the questionnaire response rate was examined, and districts with a response rate below 25% or fewer than 10 respondents were excluded on the grounds of limited representativeness (see Additional file 1). Previous research has indicated that an events-per-variable ratio of ≥10 is sufficient for logistic regression analysis (27). As a result, data from 5,964 individuals across 65 voting districts were included in the analysis. The study followed the STROBE guidelines for reporting cross-sectional observational studies (28).

Ethical approval was obtained from the Ethics Review Board of the Kyushu University Medical Division (Approval No. 29–371) on 16 November 2017. Written informed consent was obtained from all participants prior to enrolment. This research was conducted ethically in accordance with the World Medical Association Declaration of Helsinki.

### 2.2 Questionnaire content

*Basic attributes*: The basic attributes included sex and age, place of residence, academic background, economic status (ES), and the receipt of specific medical checkups. The academic background of participants was divided into four categories based on the highest level of education completed: compulsory, high school, university, or other. ES was subjectively classified into four levels, ranging from comfortable to poor. ES is an important predictor of whether a patient will undergo a medical checkup (8). The respondents were asked whether they had received a specific medical checkup in the past year by selecting “yes” or “no.”

*Social capital*: The Regional Health-Related Social Capital Indicators Version 2.0 Index was used as the social capital index (29). This index was developed in Japan, and its reliability and validity have been verified (29). The scale consisted of 11 items, divided into three subscales: civic participation, social cohesion, and reciprocity. Civic participation involved participating in five types of groups: volunteering, sports, hobbies, learning, and experiential exchanges. One point was awarded for each item for which participation occurred at least once per month. Social cohesion refers to trust in the community (“Do you think the people in your area are generally trustworthy?”), norms of reciprocity (willingness to serve others), and attachment to one’s place of residence. One point was awarded for each item for “very much” or “moderate” responses. Reciprocity indicated the exchange of emotional support (providing and receiving emotional support, such as sharing concerns or complaints) and practical support (having someone take care of you when you have been bedridden for a few days owing to illness). Regarding reciprocity, one point was awarded if such individuals were present, and −1 point was assigned if they were not (29). The scores for each item were summed to determine an individual’s level of social capital, with a higher score indicating a higher level of social cohesion. Regarding community-level social cohesion, the proportion of responses for each of the 11 indicators was calculated and aggregated for the different voting districts. To determine the regional-level social capital, response ratios for each of the 11 items were calculated on a sub-regional basis and tabulated as follows—civic participation was calculated using the formula: volunteer participation ratio × 0.6 + sports participation ratio × 0.8 + hobby participation ratio × 0.9 + learning participation ratio × 0.7 + experience transfer participation ratio × 0.5. Social cohesion was calculated as: trust ratio × 0.9 + reciprocity ratio × 0.8 + attachment ratio × 0.7. Reciprocity was calculated as: percentage of emotional support received × 0.8 + the percentage of emotional support provided × 0.7 + the percentage of instrumental support received × 0.6 (29).

### 2.3 Statistical analyses

Statistical analysis was conducted using multilevel logistic regression with data from 5,964 individuals nested within 65 voting districts. Multilevel modeling allows researchers to examine whether community-level social capital exerts an independent contextual effect and enables the investigation of cross-level interactions between community- and individual-level variables (6).

First, we estimated a null model to determine whether participation in specific health checkups varied across districts. Because inherent differences among voting districts could confound the association between community-level social capital and participation in specific health checkups, we calculated, for each district, the ageing rate and socioeconomic status (SES; percentage of those with poor economic status and compulsory education in each voting district) for Models 2–4. The models were structured as follows:

Model 1: Included only community-level social capital indicators for the 65 voting districts (community-level variables).

Model 2: Added aging rate and SES (potential confounders) to Model 1.

Model 3: Added individual social environmental factors (sex, age, ES, education level) to Model 2.

Model 4: Added individual-level social capital (civic participation, social cohesion, and reciprocity) to Model 3.

These models were used to evaluate the contextual influence of community-level social capital on individuals’ participation in health checkups. Comparisons across Models 1–4 allowed researchers to assess whether the association between community-level social capital and health checkups participation was confounded by individual-level factors. The odds ratio (OR) is a social capital parameter that is divided by the standard deviation (SD). When used for social capital, it represents the OR for a 1-SD increase. Rather than relying on post-estimation sandwich-type robust standard errors, we specified random intercepts at the voting-district level, allowing the models to structurally account for intra-cluster correlation. Although potential confounders such as community health awareness and family member support were not fully available, regional aging rate and SES proxies were included in the model.

SAS software, version 9.4 (SAS Institute Inc., Cary, NC, United States), was used for statistical analyses. A significance level of 5% was considered significant.

## 3 Results

Overall, 6,528 respondents completed the questionnaire (response rate: 49.4%). Data from 564 individuals, including those with missing data, were excluded. Table 1 presents the characteristics of the study population. Of the remaining 5,964 participants, 2,937 (49.2%) underwent specific medical checkups, and 3,026 (50.7%) did not. Among those included in the analysis, 2,649 were men and 3,315 were women, with a mean age of 69.4 years (SD = 2.8). Regarding educational background, 51.1% graduated from high school and 28.6% received only compulsory education. In terms of ES, 45.4% of the respondents said they were rather uncomfortable with their status.

**Table 1:** Population characteristics (*n* = 5,964).

Item	Values	
Age (years), *n* (%)		
65–69	3,143	52.7
70–74	2,786	46.7
Not available	35	0.6
Sex, *n* (%)		
Men	2,649	44.4
Women	3,315	55.6
Educational background, *n* (%)		
Compulsory education	1,706	28.6
High school, etc.	3,045	51.1
College, etc.	1,117	18.7
Other	63	1.1
Not available	33	0.6
Economic status, *n* (%)		
Comfortable	281	4.7
Rather comfortable	2,258	37.9
Rather uncomfortable	2,709	45.4
Poor	654	11.0
Not available	62	1.0
Specific medical checkup participation, *n* (%)		
Yes	2,937	49.2
No	3,026	50.7
Individual-level social capital, mean (SD)		
Civil participation	0.68 (1.07)	
Social cohesion	1.76 (1.13)	
Reciprocity	2.86 (0.50)	
Community-level social capital, mean (SD)		
Civic participation	51.64 (12.17)	
Social cohesion	140.58 (18.18)	
Reciprocity	200.46 (4.67)	

SD, standard deviation.

Table 2 presents the results of the multilevel logistic regression analysis examining the relationship between social capital and the presence or absence of specific medical checkups. Model 1, which only included social capital, showed higher odds of participating in specific medical checkups for each 1-SD increase in community-level social cohesion (OR = 1.11; 95% CI: 1.03–1.19). Model 2 (adjusted for aging rate and SES) and Model 3 (adjusted for sex, age, and ES) produced similar estimates, with higher levels of community-level social cohesion consistently associated with higher odds of participation. At the individual level, women, individuals with higher education, and those reporting more comfortable ES showed higher odds of participating in specific medical checkups in Models 2 and 3. In Model 4, which additionally included individual-level social capital, the association between community-level social cohesion and participation remained significant (OR = 1.11; 95% CI: 1.02–1.21), although the effect size was slightly attenuated. In this final model, women, individuals with higher education, and those with higher scores on all three individual-level social capital subscales continued to exhibit higher odds of participation, while ES was no longer associated with participation.

**Table 2:** Associations of community- and individual-level social capital with participation in specific medical checkups: results from multilevel logistic regression models.

Variables	Model 1		Model 2		Model 3		Model 4	
	OR	(95% CI)	OR	(95% CI)	OR	(95% CI)	OR	(95% CI)
Deviation	8,191.45		8,182.28		7,918.07		7,782.68	
Contextual factors								
Social capital (community-level)								
Civic participation^a^	0.99	(0.92–1.06)	0.97	(0.91–1.04)	0.97	(0.91–1.04)	0.94	(0.87–1.01)
Social cohesion^a^	1.11	(1.03–1.19)***	1.12	(1.03–1.23)***	1.14	(1.04–1.24)***	1.11	(1.02–1.21)*
Reciprocity^a^	0.99	(0.92–1.06)	0.99	(0.93–1.06)	0.99	(0.93–1.06)	0.99	(0.92–1.05)
Compositional factors								
Sex								
Women					1.00		1.00	
Men					0.71	(0.64–0.79)***	0.77	(0.69–0.86)***
Age (years)								
65–69					1.00		1.00	
70–74					1.07	(0.97–1.20)	1.02	(0.91–1.13)
Economic status								
Rather comfortable					1.00		1.00	
Rather uncomfortable					0.82	(0.73–0.91)***	0.94	(0.84–1.05)
Educational background								
Primary school or less					1.00		1.00	
High school					1.42	(1.25–1.61)***	1.31	(1.15–1.49)***
College or higher					1.44	(1.22–1.70)***	1.23	(1.04–1.46)*
Other					0.86	(0.51–1.47)	0.81	(0.47–1.40)
Social capital (individual-level)								
Civic participation^a^							1.29	(1.22–1.36)***
Social cohesion^a^							1.13	(1.07–1.19)***
Reciprocity^a^							1.13	(1.00–1.26)*
Random effects								
Variance (SE)	0.051(0.024)*		0.045 (0.023)*		0.048 (0.024)*		0.048 (0.024)*	

*p < 0.05; **p < 0.01; ***p < 0.001. Odds ratios were tested in a multi-level logistic regression analysis. ^a^ORs of social capital represent a per-standard-deviation increase of each score. Variance (SE) in the null model was 0.073 (0.028); *p* < 0.001. Model 1: community-level social capital only. Model 2: Model 1 + confounding factors for the voting district (aging rate, percentage of compulsory education, percentage of economically disadvantaged individuals). Model 3: Model 2 + individual-level social environmental factors (sex, age, socioeconomic status, education level). Model 4: Model 3 + individual-level social capital. CI, confidence interval; OR, odds ratio; SC, social capital; SE, standard error.

## 4 Discussion

### 4.1 Community-level cognitive social capital (social cohesion) and preventive health behavior

In this study, we used a multilevel model to examine whether community-level social capital defined at the voting-district level, which more closely corresponds to the typical daily living area of older adults, was associated with participation in specific medical checkups.

Even after adjusting for community-level confounders, social environmental characteristics, and other individual-level factors such as social capital, community-level social cohesion—comprising trust, social reciprocity, and attachment to one’s residential area—remained consistently associated with checkup participation. This finding is consistent with international evidence from South Korea, the United States, and China (22–24), which shows that higher community trust and reciprocity can promote the utilization of preventive health services among older populations. The present study, however, advances the literature in two important ways. First, rather than relying on broad administrative units such as municipalities or counties, we adopted voting districts as a more fine-grained and socially meaningful spatial unit that more accurately reflects the typical walking range and everyday living environment of older adults. This approach improved contextual precision and enabled a more realistic representation of neighborhood effects. Second, we clearly distinguished community-level social capital from individual-level social environmental factors and social capital and demonstrated that the former exerts an independent contextual influence even after statistically controlling for the latter.

From a theoretical perspective, our findings suggest that the social cohesion observed in this study can be understood as a core dimension of cognitive social capital—characterized by subjective perceptions of trust, reciprocity, and shared norms. In social epidemiology, such cohesion is regarded as a prerequisite for social capital to operate as a collective resource (30, 31). Communities with higher levels of cohesion are more likely to foster shared health-related norms—such as the expectation that “attending checkups is simply what one does”—thereby promoting preventive behavior through normative influence, regardless of individual motivation or resources. Moreover, when cohesion interacts with informal social control, it reinforces collective efficacy, enabling concrete forms of support; for example, ride-sharing or neighbors inviting each other to attend checkups together, which in turn lowers both psychological and practical barriers to checkup participation. Taken together, these findings suggest that social cohesion functions not merely as the aggregation of individual social ties but as an independent contextual determinant that promotes preventive health behaviors through community-level norms, trust, and collective coordination capacity.

### 4.2 Factors associated with the presence or absence of medical checkups

Consistent with previous studies, our findings showed that being female, having higher educational attainment, and possessing stronger individual-level social capital were associated with a greater likelihood of undergoing specific medical checkups. These patterns align with domestic and international evidence indicating that women and individuals with higher health literacy or stronger social connectedness are more proactive in utilizing preventive health services (7, 8, 31, 32). Regarding ES (8), an association was observed up to Model 3; however, no association was found when individual-level social capital was introduced. Therefore, the influence of individual-level social capital was deemed significant.

Importantly, the present study also demonstrated a cross-level compensation mechanism, in which community-level social cohesion offsets individual-level disadvantages. Even individuals with relatively low personal social capital were more likely to undergo medical checkups when they lived in voting districts characterized by strong community-level cohesion. This finding suggests that the social environment can support preventive health behaviors beyond individuals’ own capacity to mobilize personal resources. While individual-level social capital reflects one’s ability to draw support from personal networks, community-level cohesion may generate external contextual benefits through shared norms, neighborly encouragement, and informal support systems. These results highlight that individual agency is not exercised in isolation but is shaped—and in some cases actively reinforced—by the broader community context.

### 4.3 Limitations and future studies

When interpreting the findings of this study, some limitations should be acknowledged. First, because the study employed a cross-sectional design, causal relationships cannot be established, and the potential for reverse causality or self-selection cannot be completely ruled out. For instance, individuals who are already motivated to undergo health checkups may be more inclined to participate in community activities, which in turn could strengthen local social cohesion. Although we partially addressed this concern by adjusting for potentially influential contextual factors, future research should adopt longitudinal designs to more rigorously identify causal pathways. Moreover, given the response rate of 49.4%, respondents may have been disproportionately healthier older adults capable of completing and returning the questionnaire, which could introduce additional selection bias.

Second, although we controlled for regional socioeconomic structure using voting district–level aging rates and SES, unobserved contextual confounders, such as accessibility to specific medical checkup sites and local transportation infrastructure, may not have been fully captured. Future studies should address this limitation by applying alternative causal inference approaches, such as propensity score matching or marginal structural models, to more rigorously examine causal relationships. Additionally, because individual-level economic status was assessed using self-reported subjective perceptions, measurement bias may exist; future research should consider incorporating more objective socioeconomic indicators where feasible.

Third, this study targeted the entire population of adults aged 65–75 years residing in a single city, with voting districts covering geographically diverse areas, including coastal, mountainous, and central zones. However, we did not examine whether the observed associations differed across major subgroups, such as coastal, mountainous, and central urban areas, or according to levels of transportation accessibility. Future research should explore such potential heterogeneity to enhance the robustness and generalizability of the findings.

Finally, the study was conducted in a single local municipality in Japan, and although the use of fine-grained voting districts improves contextual precision, the generalizability of the findings should be validated in other cultural and institutional settings. Nevertheless, given that rapid population aging and declining community ties are common challenges in many societies, our results likely hold broader implications beyond the Japanese context.

### 4.4 Policy implications

The findings of this study suggest that strengthening cognitive social capital within micro-scale community units, such as voting districts that lie within walking distance for older adults, may serve as an important complementary strategy to conventional individual-focused health promotion policies. In rapidly aging societies, approaches that target only older individuals may be insufficient; instead, interventions that foster collective norms, promote informal neighborly support, and cultivate socially cohesive environments may represent effective strategies for increasing participation in specific health checkups.

Practically, this could involve establishing neighborhood-based health promotion platforms, such as small-scale community gatherings, home visits by public health nurses, or peer-led networks that encourage checkup participation. Importantly, these interventions do not necessarily require large financial resources; rather, by strategically leveraging existing social relationships and community trust, they may help normalize checkup participation as a shared social expectation. From a policy perspective, focusing on submunicipal units such as voting districts enables more precise identification of areas with weak social cohesion and low screening uptake, thereby allowing for more strategic intervention design.

Given that population aging and the weakening of community connectedness are global phenomena, these implications extend beyond the Japanese context. Particularly in settings where health systems increasingly rely on preventive participation to control costs and promote healthy longevity, building socially cohesive microcommunities may represent a scalable and culturally adaptable strategy across diverse national contexts.

## Data Availability

The data that support the findings of this study are available from the corresponding author, HM, upon reasonable request.
